# Treatment with Tacrolimus and Sirolimus Reveals No Additional Adverse Effects on Human Islets *In Vitro* Compared to Each Drug Alone but They Are Reduced by Adding Glucocorticoids

**DOI:** 10.1155/2016/4196460

**Published:** 2016-01-18

**Authors:** Kristine Kloster-Jensen, Afaf Sahraoui, Nils Tore Vethe, Olle Korsgren, Stein Bergan, Aksel Foss, Hanne Scholz

**Affiliations:** ^1^Department of Transplant Medicine, Oslo University Hospital, P.O. Box 4950, 0424 Oslo, Norway; ^2^Institute for Surgical Research, Oslo University Hospital, P.O. Box 4950, 0424 Oslo, Norway; ^3^Institute of Clinical Medicine, University of Oslo, P.O. Box 1171, Blindern, 0318 Oslo, Norway; ^4^Department of Pharmacology, Oslo University Hospital, P.O. Box 4950, 0424 Oslo, Norway; ^5^Science for Life Laboratory, Department of Immunology, Genetics and Pathology, Uppsala University, Box 815, 75108 Uppsala, Sweden; ^6^Department of Clinical Immunology, Genetics and Pathology, Rudbeck Laboratory, Uppsala University Hospital, 75185 Uppsala, Sweden; ^7^School of Pharmacy, University of Oslo, P.O. Box 1171, Blindern, 0318 Oslo, Norway

## Abstract

Tacrolimus and sirolimus are important immunosuppressive drugs used in human islet transplantation; however, they are linked to detrimental effects on islets and reduction of long-term graft function. Few studies investigate the direct effects of these drugs combined in parallel with single drug exposure. Human islets were treated with or without tacrolimus (30 *μ*g/L), sirolimus (30 *μ*g/L), or a combination thereof for 24 hrs. Islet function as well as apoptosis was assessed by glucose-stimulated insulin secretion (GSIS) and Cell Death ELISA. Proinflammatory cytokines were analysed by qRT-PCR and Bio-Plex. Islets exposed to the combination of sirolimus and tacrolimus were treated with or without methylprednisolone (1000 *μ*g/L) and the expression of the proinflammatory cytokines was investigated. We found the following: (i) No additive reduction in function and viability in islets existed when tacrolimus and sirolimus were combined compared to the single drug. (ii) Increased expression of proinflammatory cytokines mRNA and protein levels in islets took place. (iii) Methylprednisolone significantly decreased the proinflammatory response in islets induced by the drug combination. Although human islets are prone to direct toxic effect of tacrolimus and sirolimus, we found no additive effects of the drug combination. Short-term exposure of glucocorticoids could effectively reduce the proinflammatory response in human islets induced by the combination of tacrolimus and sirolimus.

## 1. Introduction

Despite promising results in islet transplantation, glycaemic control is gradually impaired due to progressive graft dysfunction [1, 2]. The initial loss of islets immediately after islet transplantation is a result of inflammatory events and an alloantigen-nonspecific inflammatory process where inflammatory cytokines play a part in cellular injury to islets. Another major reason for graft dysfunction is toxicity caused by the use of immunosuppressive drug therapy. The common immunosuppressive protocols used in clinical islet transplantation include induction therapy with either ATG or IL-2 receptor monoclonal antibody, followed by maintenance treatment including that of tacrolimus and sirolimus [1]. Tacrolimus, a CNI (calcineurin inhibitor), is the pillar of immunosuppressive therapy in both solid organ and islet transplantation because of its efficacy in preventing acute rejection and improving short-term graft survival [1, 3]. Tacrolimus inhibits insulin secretion and reduces islets viability [4–6]. Sirolimus is an mTOR (mammalian target of rapamycin) inhibitor, which inhibits cell proliferation and reduces allograft rejection resulting in improved long-term graft survival [7, 8]. Sirolimus decreases insulin secretion in islets, impairs revascularization, and reduces angiogenesis [9–11]; however the impact of these effects on transplanted islets is not fully characterized [12]. To what extent the combination treatment of sirolimus and tacrolimus shows additive toxicity to islets is not fully elucidated and few studies compare the direct effects on human islets of this drug combination* in vitro* [13, 14].

The instant inflammatory reaction observed in the peritransplant period after human islet transplantation leads to loss of more than 50% of the injected islets within the first hour [2, 15, 16]. Several approaches such as inhibition of specific cytokines such as TNF alpha and IL-1beta have been studied to evaluate potency in helping reduce this effect [17, 18]. Glucocorticoids are well-known immunosuppressive agents in transplantation, with strong anti-inflammatory properties by inhibiting production of several cytokine and chemokine. But because glucocorticoids have diabetogenic effects* in vivo* and directly impair insulin secretion, they have mostly been excluded from the immunosuppressive regimens in clinical islet transplantation after presentation of the Edmonton protocol. We have previously shown that short-term use of methylprednisolone during islet culturing prior to transplantation is effective for maintaining islet viability by reducing the proinflammatory cytokines production even though insulin secretion was temporarily suppressed [19].

This study was undertaken to elaborate on the direct effects of the combination treatment with tacrolimus and sirolimus on the function and viability in human islets compared to the effect of each drug alone. Finally we also investigated the effect of methylprednisolone, a glucocorticoid, on human islets after treatment with the combination of tacrolimus and sirolimus.

## 2. Materials and Methods 

### 2.1. Islet Isolation and Culture

Human islets were isolated from 5 human pancreata obtained from multiorgan donors (one female and four males) after appropriate consent in the islet isolation laboratory facility at The Nordic Network for Clinical Islet Transplantation, Uppsala University Hospital, Sweden, according to the automated method refined by the Nordic Network for Islet Transplantation [20]. Approval of the experimental use of the islets was granted by the local Institutional Ethical Committee and performed in accordance with the principles of the Declaration of Helsinki 2000. The average donor age was 53 years (range 39–60 years), the body mass index (BMI) 26.6 kg/m^2^ (range 22–32 kg/m^2^). All donors met the criteria with glycosylated haemoglobin A1c below 6.5% (48 mmol/mol) [21]. Islet preparations were maintained in culture medium CMRL1066 (Mediatech), supplemented with 10% ABO-compatible serum, 10 mM Hepes, and 1% penicillin/streptomycin/L-glutamine (Invitrogen), at 37°C (5% CO_2_) for the first 24 hours after isolation. After a medium change, the islets were maintained at 22°C (5% CO_2_) until being used in experiments.

### 2.2. Immunosuppressive Drugs and Culturing

Between 2 and 5 days after isolation, equal aliquots of clinical grade islet preparations (purities from 75 ± 20% and viability of 85 ± 5%) were placed into 90 mm Petri dishes and cultured with tacrolimus 30 *µ*g/L (37 nM), sirolimus 30 *µ*g/L (33 nM) (Sigma-Aldrich, St. Louis, MO), or the combination thereof for 24 hours at 37°C (5% CO_2_). Each experiment comprised control and stimulated islets from the same donor. In parallel experiments methylprednisolone (1000 *μ*g/L) was added or not to the combined treatment of tacrolimus and sirolimus for 24 hours at 37°C (5% CO_2_). We used our previous investigation into the blood through concentrations of methylprednisolone after a bolus dose of 500 mg intravenously in liver transplant recipients, to select the dose to use in* in vitro* exposure [22, 23]. We have also shown that 48 hours use of different doses of methylprednisolone to human islets* in vitro* reduced the viability and insulin secretion, without representing a durable detrimental effect [19]. Many transplant centers include a single dose of methylprednisolone as premedication prior to islet transplant [24]. Based on these findings, we selected 100 mg as the dose for the present study in order to investigate the anti-inflammatory effects of methylprednisolone on immunosuppressive exposed human islets.

Following treatment, cultured supernatants were collected and human islets were hand-picked into columns, washed two times with ice-cold phosphate buffered saline (PBS) before being used for either RNA extraction or lysed in 200 *μ*L MiliQ water before homogenization by sonication, and then stored at −70°C until analyses with RT-PCR or multiplex bioassay (Bio-Plex human cytokine assay), respectively. The drug concentrations were selected to simulate toxic blood drug concentrations observed in portal vein immediately after transplantation [25]. Generally the therapeutic level of both FK506 (Tacrolimus) and rapamycin (Sirolimus) is assumed to be between 5 and 15 *μ*g/L, although it may reach toxic levels of 20–25 *μ*g/L [26]. Animal studies have shown that portal vein peak concentrations after 12 hours of tacrolimus and sirolimus are 5–7 times higher than the mean 24 hours through systemic level [25]. To represent a high level of drug concentration we therefor chose to use 30 *μ*g/L. All drugs were solved in methanol and diluted in cell culture medium to reach their final concentrations.

### 2.3. Glucose-Stimulated Insulin Secretion Assay

Following treatment, twenty islets were hand-picked, transferred into 12 Transwell trays (Costar, Cambridge, MA, USA), and preincubated in Krebs-Ringer bicarbonate buffer (11.5 mM NaCl, 0.5 mM KCl, 2.4 mM NaHCO_3_, 2.2 mM CaCl_2_, 1 mM MgCl_2_, 20 mM HEPES, and 2 mg/L albumin: all Sigma-Aldrich) containing 1.67 mmol/L glucose (Fresenius Kabi, Halden, Norway) at 37°C (5% CO_2_) for 30 min before the islets were incubated for 40 min in fresh Krebs-Ringer bicarbonate buffer containing 1.67 mmol/L glucose (basal insulin secretion). Finally, the islets were incubated for 40 min in fresh Krebs-Ringer bicarbonate buffer containing 20.0 mmol/L glucose (for stimulated insulin secretion). The supernatants were subsequently collected, and insulin concentration was measured using a human insulin enzyme immunoassay (EIA) (Mercodia AB, Uppsala, Sweden). Stimulation index (SI) expresses the islets capacity for insulin secretion and is calculated as the ratio of insulin secretion at 20.0 mmol/L to 1.67 mmol/L glucose/40 min.

### 2.4. Detection of Cell Apoptosis and Cell Death

Assessment of apoptosis in islets was measured by the detection of DNA-histone complexes present in the cytoplasmic fraction of the cells using Cell Death Detection ELISA^PLUS^ (Roche, Basel, Switzerland) according to the instructions of the manufacturer.

### 2.5. Real-Time Quantitative PCR

Total RNA was isolated from frozen islet pellets using the RNeasy Mini Kit (Qiagen, Hilden, Germany) according to manufacturer's guidelines. The concentration of all RNA samples was quantified using a NanoDrop ND-1000 UV/Vis spectrophotometer (Saveen Werner AB, Sweden), and 1 *μ*g total RNA was reverse-transcribed using the High-Capacity cDNA Archive Kit according to the instructions of the manufacturer (Applied Biosystems, Forster City, CA, USA). Quantification of mRNA expression was performed using the following TaqMan assays: human IL-1*β*: Hs00174097m1 and IL-8: Hs00174103m1 with an ABI 7900HT Fast Real-Time PCR System (Applied Biosystems). Results were normalised to the housekeeping gene beta-actin and data were analysed using the 2^−ΔΔCt^ method.

### 2.6. Measurement of Proinflammatory Cytokines

Concentrations of IL-8 and IL-6 were measured in islet lysate utilizing multiplex technology on a Multiplex Analyser (BioRad, Hercules, CA) following the instructions of the manufacturer. Each sample was correlated to the protein content of the islet lysate measured by protein assay kit (BCA; Pierce, Rockford, IL, USA).

### 2.7. Statistical Analyses

Results are presented as mean ± SEM. Statistical analyses were performed with Kruskal-Wallis one-way analysis of variance (ANOVA) followed by unpaired *t*-test. Differences were considered significant at levels of *p* < 0.05. Statistical analyses were performed using GraphPad Prism 5.0 (GraphPad Software, CA, USA).

## 3. Results 

### 3.1. No Additive Adverse Effect on Human Islet Function by the Combination Treatment with Tacrolimus and Sirolimus Compared to Tacrolimus Alone

**Figure 1:**
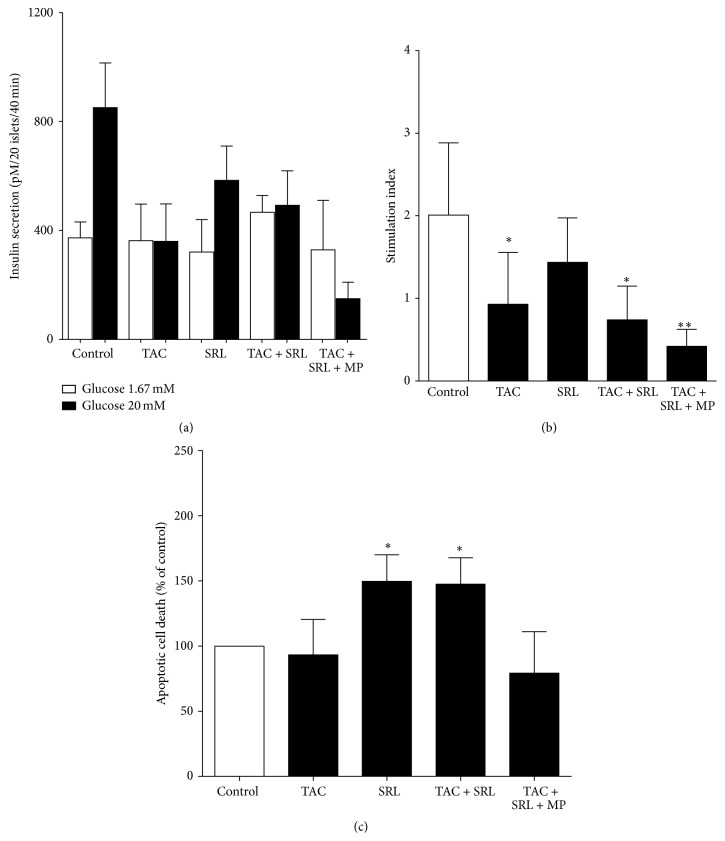
The effect of immunosuppressant drugs on human islets function and survival. Human islets treated without or with tacrolimus (TAC 30 *µ*g/L), sirolimus (SRL 30 *µ*g/L), or a combination thereof with or without methylprednisolone (MP 100 ng/L) for 24 h. (a) Glucose-stimulated insulin secretion (GSIS) and (b) corresponding stimulation index (SI) (ratio stimulated to basal glucose-stimulated insulin secretion) in human islets as described in Section 2. (c) Apoptotic cell death measured by the Cell Death Detection ELISA^PLUS^ in human islet following the same culture conditions. Data are presented as means ± SEM of five separate experiments from five different donors. ^*∗*^ *p* < 0.05, ^*∗∗*^ *p* < 0.006 versus control.

To investigate the effect of tacrolimus and sirolimus on human islets function compared to either drug alone, we performed a glucose challenge test after 24 hours of exposure to either tacrolimus or sirolimus or the combination thereof. The insulin release from control islets was significantly increased in response to stimulation with high glucose (20 mM) solution compared to low glucose (1.67 mM) solution (Figure 1(a)). The combination treatment of tacrolimus and sirolimus resulted in a slight increase of basal insulin secretion and a reduced glucose-stimulated insulin secretion after stimulation with high glucose solution (Figure 1(a)). These results lead to a significant reduction of the stimulation index (SI) compared to untreated islets (*p* = 0.0193; Figure 1(b)). When methylprednisolone was added to the combination treatment we found no altered basal insulin secretion level in human islets after incubation in low glucose solution, whereas we found reduced insulin secretion after 1 hour in high glucose solution (Figure 1(a)), which led to a reduced SI (*p* = 0.0057; Figure 1(b)). As previously reported, tacrolimus treated islets show a significant reduction in capacity for stimulated insulin secretion (Figure 1(a)) [4] with no effects on the insulin content [5]. This suppression of high glucose-induced insulin release is also expressed by the reduced stimulation index compared to untreated control (*p* = 0.0400; Figure 1(b)). Following the incubation with sirolimus alone, the islets presented an almost normal insulin secretion both at basal and at stimulated level (*p* = 0.273), in accordance with previous findings [12]. Importantly when we compared the effects of the combination to each drug alone, we did not find additional deleterious effects of the combined treatment compared to tacrolimus alone on the GSIS in human islets (Figures 1(a) and 1(b)).

### 3.2. The Combination Treatment of Tacrolimus and Sirolimus Induced Apoptosis in Human Islets

In order to investigate its potential role in the reduction of islets function, we characterized apoptosis by measurements of the double-stranded DNA breaks using Cell Death ELISA^PLUS^. Human islets treated with the combination of tacrolimus and sirolimus or sirolimus alone showed a significantly increased apoptosis (*p* < 0.05; Figure 1(c)) whereas tacrolimus exposure alone did not affect the apoptosis. In addition, the combination of tacrolimus and sirolimus with methylprednisolone showed no significant difference compared to untreated controls suggesting that methylprednisolone helps reduce apoptosis in islets exposed to the drug combination.

### 3.3. Treatment with Tacrolimus and Sirolimus Induces Expression of Proinflammatory Cytokines

**Figure 2:**
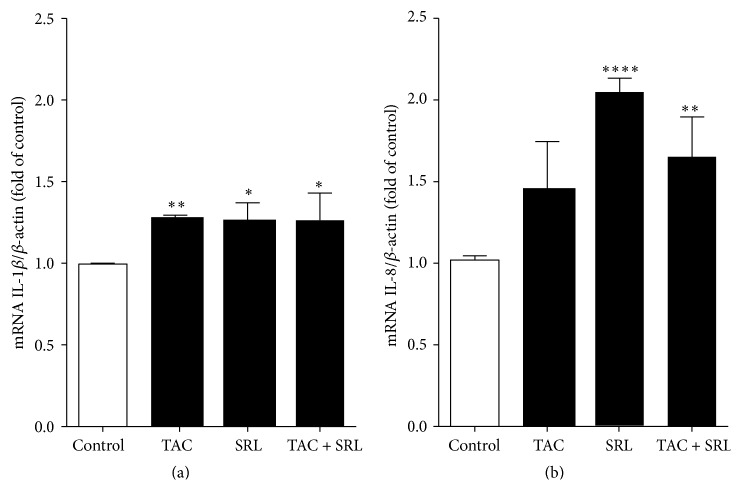
The effect of immunosuppressant drugs on gene expression of IL-1beta and IL-8 in human islets. Human islets cultured without or with tacrolimus (TAC 30 *µ*g/L), sirolimus (SRL 30 *µ*g/L), or a combination thereof for 24 hours were tested for gene expression of (a) IL-1beta and (b) IL-8 by qPCR in relation to the control gene *β*-actin. Data are presented as mean ± SEM of four separate experiments from four different donors. ^*∗*^ *p* < 0.05, ^*∗∗*^ *p* < 0.005, and ^*∗∗∗∗*^ *p* < 0.0001 versus control.

**Figure 3:**
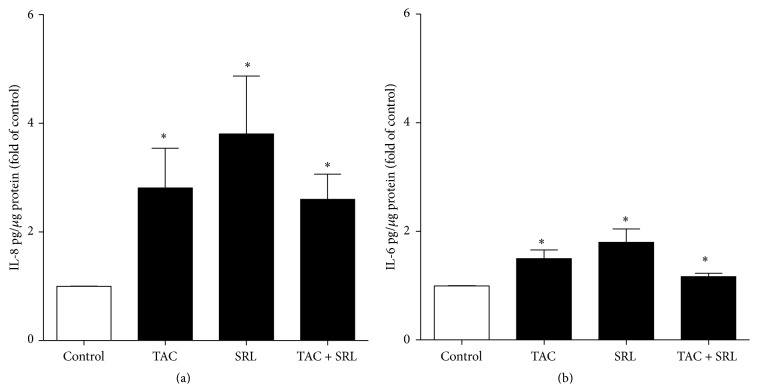
The effect of immunosuppressant drugs on protein release of IL-8 and IL-6 in human islets. Human islets cultured without or with tacrolimus (TAC 30 *µ*g/L), sirolimus (SRL 30 *µ*g/L), or a combination thereof for 24 hours. Protein levels of (a) IL-8 and (b) IL-6 in human islets were investigated in cell lysate using multiplex bead-based cytokine assay (Bio-Plex Human Cytokine Group 1). Data is presented as mean ± SEM of four separate experiments from four different donors. ^*∗*^ *p* < 0.05 versus control.

To further characterize the molecular mechanisms associated with the detrimental effect of tacrolimus and sirolimus, we studied the expression (mRNA and protein) of the proinflammatory cytokines, IL-8, IL-6, and IL-1beta, known mediatorscausing pancreatic islet dysfunction and apoptosis [27, 28]. We found increased mRNA expression levels of IL-1beta (Figure 2(a)) and IL-8 (Figure 2(b)) in human islets after exposure to the combined treatment with tacrolimus and sirolimus as well as each of the drugs alone, compared to untreated controls. However, no additive elevation of the mRNA expression of neither IL-1beta (Figure 2(a)) nor IL-8 (Figure 2(b)) in the combination treatment compared to either drug alone was observed. Correspondingly, we found a significant increased protein expression of IL-8 (Figure 3(a)) and IL-6 (Figure 3(b)) in human islets.

### 3.4. Methylprednisolone Reduce the Proinflammatory Response Induced by the Combined Treatment with Tacrolimus and Sirolimus

**Figure 4:**
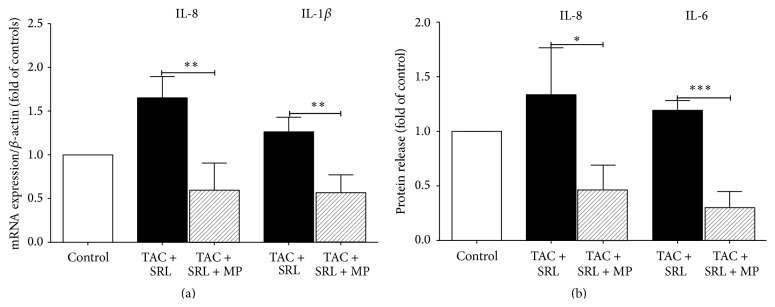
The effect of methylprednisolone on the inflammatory potential in human islets. Human islets cultured with tacrolimus (TAC 30 *µ*g/L), sirolimus (SRL 30 *µ*g/L), with or without methylprednisolone (MP 1000 ng/L), for 24 hours were tested for (a) gene expression of IL-1beta and IL-8 and (b) protein release of IL-8 and IL-6. The fold change represents the expression of target genes in isolated islets relative to the *β*-actin mRNA level. Data is presented as mean ± SEM of four separate experiments from four different donors. ^*∗*^ *p* < 0.05, ^*∗∗*^ *p* < 0.005, and ^*∗∗∗*^ *p* < 0.0005 versus control.

Adding methylprednisolone to the combination treatment caused a significant reduction in gene expression of IL-8 (*p* = 0.0023) and IL-1beta (*p* = 0.0027) (Figure 4(a)) in the human islets. This reduction was followed by significantly reduced protein levels of IL-8 (*p* = 0.0115) and IL-6 (*p* = 0.0001) in human islets compared to the combination alone (Figure 4(b)).

## 4. Discussion 

Many studies compare the direct effects of immunosuppressive drug regimens on islets* in vitro*, but few are on human islets. And although a wide range of clinical observations involve drug regimens few studies compare the effects of regimens to the effects of the drugs alone [1, 29, 30].

In this study we investigate the impact of the combined drug therapy of tacrolimus and sirolimus on islets compared to the exposure to each drug alone. Also, we explore how this drug combination is influenced by methylprednisolone. In summary, we found that the combination of tacrolimus and sirolimus does not reduce human islet function and survival more than each of the drugs alone, nor does it further increase proinflammatory cytokine expression. Methylprednisolone reduced the proinflammatory cytokine expression induced by the combination of tacrolimus and sirolimus.

In our data islets function was reduced when exposed to tacrolimus whereas sirolimus exposure did not significantly influence islet function. This is consistent with clinical observations where islets cultured with the combination tacrolimus and sirolimus decrease GSIS [31, 32]. Despite different mechanisms of action, tacrolimus [4, 6] and sirolimus [12, 31, 33, 34] ultimately reduced islet function. Sirolimus is known to influence glucose homeostasis through reduction in mitochondrial ATP production, decrease in beta cell proliferation, and impairing of insulin secretion and resistance as a consequence of chronicle exposure [11, 35]. Since our study was an acute* in vitro* study it is likely that deteriorating effects of sirolimus would be masked by the short exposure time.

Apoptosis is a major cause of islet cell loss in the early posttransplantation period [36] and it is triggered by the isolation process, islet hypoxia [37], and proinflammatory cytokines [38]. Human islets exposed to either sirolimus or tacrolimus have been shown to induce apoptosis in human islets regardless of added dose [4, 9, 29]. But tacrolimus is also shown not to cause significant apoptosis in beta-cells [6, 39], which correlates well with our findings of no increase in cell death following tacrolimus exposure. Short time exposure of tacrolimus has recently been shown to highly suppress insulin secretion in human islets without changing intracellular insulin content or viability [40]. This is consistent with our study (Figure 1(c)) and others [6, 39]. Sirolimus reduced the survival of islets, supporting the findings from previous studies showing that sirolimus conclusively causes detrimental effects on beta-cells survival and cell apoptosis both in murine and in human beta-cells [9, 41] and increased apoptosis [9, 29].

Following islet transplantation proinflammatory cytokines such as IL-1beta and IL-8 are activated [42] suggesting an alloantigen nonspecific, inflammatory process [42, 43]. We observed a significantly increased expression of IL-1beta, IL-8, and IL-6 on mRNA and protein levels in islets exposed to tacrolimus and sirolimus equally in the combination and single drugs. Our observation could therefore illustrate a reciprocal inhibition of the two drugs [13] or that maximum intervention has been reached. Others have shown that sirolimus and tacrolimus have anti-inflammatory properties [44–47]. Compared to our data they use longer exposure time and lower drug dose, which are reasonable explanations for these differences.

Tacrolimus and sirolimus are structurally similar and both bind to FK506 binding proteins to form immunosuppressive complexes; however they are not considered antagonistic to one another [48]. We found no significant difference in impact on islets exposed to the drug combinations compared to each drug alone. One explanation for this could be that tacrolimus and sirolimus exert opposite effects and inhibit the actions of each other [13, 14, 49]. Another explanation could be that both drugs act by means of a common immunosuppressant binding protein or yet unidentified intracellular proteins. We recently published a paper where we found a difference in intracellular uptake of drugs when tacrolimus and sirolimus are given in combination compared to separately [50]. Even though it has been debatable we cannot exclude an antagonism between tacrolimus and sirolimus [48, 51] or that there is a distinction between high and low dose being essential for the drug-drug interaction [52].

It is well known that corticosteroid induces hyperglycemia mainly by reducing insulin-mediated glucose uptake. But the direct beta-cells toxicity through inhibition of insulin production and secretion is still controversial; most likely it depends on dose and exposure time [19, 53]. Improvement in the outcome of islet transplantation after the Edmonton protocol is partially due to steroid-free immunosuppressive protocol [1]. To enhance therapeutic efficacy while minimizing the toxicity of each drug multiple immunosuppressive drugs are being used in transplantation. Due to proinflammatory cytokines impact on islet survival, induction therapy has shifted from interleukin-2 receptor antagonist alone to a variety of new T-cell depleting antibodies, TNF alpha inhibitors, CXCR1/2 blocker, and several others [1, 54]. Our findings support that induction treatment may be needed to reduce inflammatory reactions during transplantation. We found that methylprednisolone reduced cytokine and chemokine production from islet preparations; this accords well with previous findings [55, 56]. On the other hand steroids are also known to reduce insulin secretion from beta-cells [57]; fortunately this is not permanent state [19] and a brief exposure to steroids in the preculturing phase even improves survival in an experimental mouse model. The proinflammatory cytokine production caused by sirolimus and tacrolimus was suppressed by methylprednisolone. Because of their diabetogenic properties it is still controversial whether glucocorticoids are deleterious to islets in the acute phase of transplantation. Indeed, a new trend according to the latest CITR report demonstrates that glucocorticoids are used after first infusion in approximately 17% of islet transplant patients as part of the immunosuppressive treatment, while at 6 months only 6% use these drugs [1]. Our results support short-term use of methylprednisolone as an inhibitor of the cytokine release following tacrolimus and sirolimus.

In conclusion, the* ex vivo* exposure of human islets to tacrolimus, sirolimus, and the combination of the two reduces islet function and survival and increases proinflammatory cytokines in the islets. The proinflammatory response of the tacrolimus-sirolimus combination was reduced by short-term exposure to methylprednisolone. These findings provide a basis for further investigations into the drug interactions between tacrolimus and sirolimus that can help tailor immunosuppressive regimens for islet transplant recipients.

## Abbreviations

CNI: Calcineurin inhibitor
GSIS: Glucose-stimulated insulin secretion.
